# Robust Reconstruction of Electrocardiogram Using Photoplethysmography: A Subject-Based Model

**DOI:** 10.3389/fphys.2022.859763

**Published:** 2022-04-25

**Authors:** Qunfeng Tang, Zhencheng Chen, Yanke Guo, Yongbo Liang, Rabab Ward, Carlo Menon, Mohamed Elgendi

**Affiliations:** ^1^ School of Electronic Engineering and Automation, Guilin University of Electronic Technology, Guilin, China; ^2^ Department of Electrical and Computer Engineering, University of British Columbia, Vancouver, BC, Canada; ^3^ Biomedical and Mobile Health Technology Laboratory, Department of Health Sciences and Technology, Zurich, Switzerland

**Keywords:** digital health, data science, intensive and critical care, cardiology, electrocadiogram, vital sign analysis

## Abstract

Electrocardiography and photoplethysmography are non-invasive techniques that measure signals from the cardiovascular system. While the cycles of the two measurements are highly correlated, the correlation between the waveforms has rarely been studied. Measuring the photoplethysmogram (PPG) is much easier and more convenient than the electrocardiogram (ECG). Recent research has shown that PPG can be used to reconstruct the ECG, indicating that practitioners can gain a deep understanding of the patients’ cardiovascular health using two physiological signals (PPG and ECG) while measuring only PPG. This study proposes a subject-based deep learning model that reconstructs an ECG using a PPG and is based on the bidirectional long short-term memory model. Because the ECG waveform may vary from subject to subject, this model is subject-specific. The model was tested using 100 records from the MIMIC III database. Of these records, 50 had a circulatory disease. The results show that a long ECG signal could be effectively reconstructed from PPG, which is, to our knowledge, the first attempt in this field. A length of 228 s of ECG was constructed by the model, which was trained and validated using 60 s of PPG and ECG signals. To segment the data, a different approach that segments the data into short time segments of equal length (and that do not rely on beats and beat detection) was investigated. Segmenting the PPG and ECG time series data into equal segments of 1-min width gave the optimal results. This resulted in a high Pearson’s correlation coefficient between the reconstructed 228 s of ECG and referenced ECG of 0.818, while the root mean square error was only 0.083 mV, and the dynamic time warping distance was 2.12 mV per second on average.

## Introduction

Cardiovascular disease is a major cause of health loss that could be preventable (Thomas, 2018). Long-term monitoring of cardiovascular status is of great significance in preventing and treating cardiovascular diseases. Electrocardiography is widely used in cardiovascular diagnoses and cardiovascular status monitoring in clinical practice worldwide. The morphological features of an electrocardiogram (ECG) (e.g., P, Q, R, S, T and the intervals between them) can usually enable the diagnosis of abnormalities of the heart (Rai et al., 2013; Joshi et al., 2014). However, most conventional ECG devices limit subjects’ movements, to prevent ECG electrodes from detaching. To address the shortcomings of traditional devices for long term ECG monitoring, wearable ECG devices have been designed in recent years (Steinberg, 2019; Marsili, 2020). Meanwhile, another technology that can detect cardiovascular status without the need for electrodes, called photoplethysmography, has flourished in the last decade (Elgendi, 2020). Photoplethysmograms (PPGs, which is the recommended acronym based on this study (Elgendi, 2012a)) are more comfortable and less expensive to obtain than ECG signals, and PPG is expected to be an alternative in cardiovascular status monitoring (Elgendi, 2012b). In fact, recent research has shown that the ECG features are not consistently correlated with blood pressure (Bird, 2020), while PPG features are consistently correlated with blood pressure (Elgendi, 2019; Hosanee, 2020).

**FIGURE 1:**
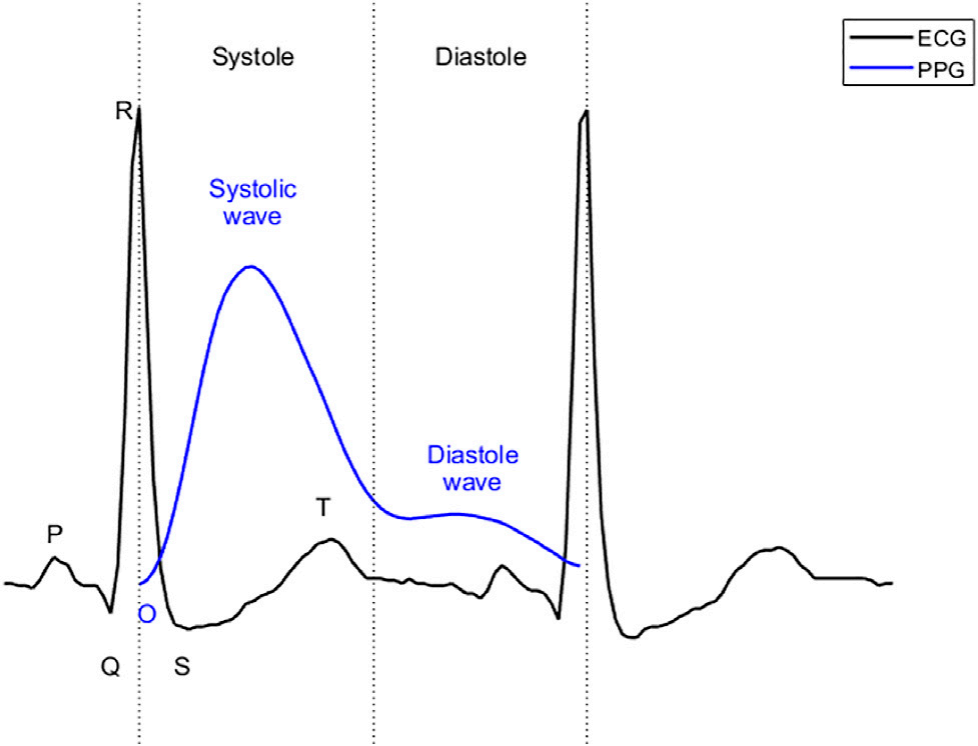
Systole and diastole in ECG and PPG. The “O″” stands for the onset in PPG.

ECG reflects the circulatory electrical activity of the heart. Each heartbeat is triggered by electrical impulses, typically generated by the sinoatrial node and transmitted throughout the heart. At specific points during the heart’s electrical beat, the ECG records the sum of the many weak fields generated by the electrical activity of many heart cells. The electrical activity of the heart causes systole and diastole through excitation-contraction coupling (Bers, 2002). The heart contracts and relaxes periodically, thus serving as an engine that pumps blood through the body. For systemic circulation, blood is pumped from the left ventricle to the main artery and through the arterial branches to the capillaries of the organs. The blood then passes through the capillary walls, exchanging substances and gases with tissue cells, facilitated by tissue fluid. After the exchange, arterial blood becomes venous blood and flows back to the right atrium through various veins. PPG uses optical technology to record changes in blood volume at body peripherals (e.g. fingertips, ears, and wrists) (Elgendi, 2012b; Hosanee, 2020). This change is a result of the systemic circulation. As shown in Figure 1, in ECG, the beginning of systole is marked by the QRS complex, while the beginning of diastole is marked by the end of the T wave. For PPG, the beginning of systole is marked by onset, the first wave (systolic wave) reflecting the systole, and the second wave (diastolic wave) representing the diastole. Since it takes time for blood to travel from the heart to the end of the body, the onset of the PPG is delayed relative to the R peak of the ECG. This delay is called the pulse arrival time (Liang, 2019). Pulse arrival times have been removed in Figure 1 to align systole in ECG with that in PPG. The morphology of PPG is affected by many factors, such as the mechanical movement of the heart, and the compliance state of blood vessels during blood transmission. Therefore, PPG contains a wealth of information regarding the cardiovascular system. Recently, PPG has been used to evaluate cardiovascular parameters such as heart rate, oxygen saturation (Shelley, 2007), blood pressure (Liu, 2019), and cardiac output (Wang et al., 2009).

Some studies have explored the relationship between ECGs and PPGs. Heart rate variability measured by PPG is highly correlated with that measured by an ECG (Weinschenk et al., 2016). In other words, the periodicity of PPGs is highly correlated with that of ECGs. Moreover, they are particularly correlated when arrhythmia occurs (Polanía et al., 2015; Paradkar and Chowdhury, 2017). In terms of the characteristics of a heartbeat, some important parameters of an ECG are also related to a PPG (Banerjee et al., 2014). The PR, QRS, QT and RR intervals can be estimated from features in the PPG. If a PPG can be used to synthesize an ECG, we can use both the rich clinical knowledge of signals detected in an ECG and the accessibility of PPG signals to better evaluate cardiovascular status.

The notion of generating ECG signals has been discussed and investigated in the literature, with great emphasis on understanding and modeling waveform morphologies (McSharry et al., 2003; Sayadi et al., 2010). Statistical modeling is generally used to generate a beat by beat synthetic ECG signal (usually RR intervals). The ECG beats are then stitched together consecutively based on beat information. Some researchers have employed three-lead ECG to reconstruct 12-lead ECG (Maheshwari et al., 2014) and compressive sensing ECG reconstruction (Craven et al., 2017). Typically, the construction of ECG does not depend on another physiological signal collected at the same time.

To our knowledge, there are two papers that have reconstructed an ECG from a PPG recorded simultaneously (Tian et al., 2020; Zhu et al., 2021). One of them is a beat-based model (Zhu et al., 2021). The study developed a linear transformation model to reconstruct ECG signals. Discrete cosine transform (DCT) coefficients for each PPG beat were mapped to coefficients for the corresponding ECG beat according to their proposed cardiovascular signaling model. The other work developed a cross-domain joint dictionary learning (XDJDL) model to establish a mapping relationship between PPG and ECG beats (Tian et al., 2020). However, the accuracy of these two methods depends on the accuracy of extraction algorithms for R waves in an ECG and systolic peak (or onset) in a PPG, which makes the algorithm more complex and introduces greater opportunities for error, thus decreasing the ECG reconstruction’s accuracy. Moreover, these two studies were conducted when the training set and the test set accounted for 80% and 20% of the data set, respectively. Their long-term performance on data longer than the training set has not been verified.

This paper proposes a deep neural network model that constructs long ECG signal from PPG. Unlike prior works, it has the advantage of not requiring R-peak detection or a beat-based ECG segmentation step prior to the analysis. It aims to mitigate the errors introduced by period division and to evaluate the performance of the model in generating long ECG signal.

## Methodology

### Dataset

The data we used to test the model was extracted from the MIMIC III matched subset (Johnson, 2016). The MIMIC III database contains several physiological signals from intensive care unit patients. And there are a large number of records in this subset. We used 100 records (50 subjects with circulatory diseases and 50 subjects without) of different subjects with lead II ECG and PPG signals. Every record was 5 min in length. Note that both ECG and PPG were recorded simultaneously. The reason behind the choice of 100 records is to stay consistent with the number of recordings used in the literature (Zhu et al., 2021). This will allow us to have a fair evaluation of all algorithms used.

### Proposed Method

**FIGURE 2:**
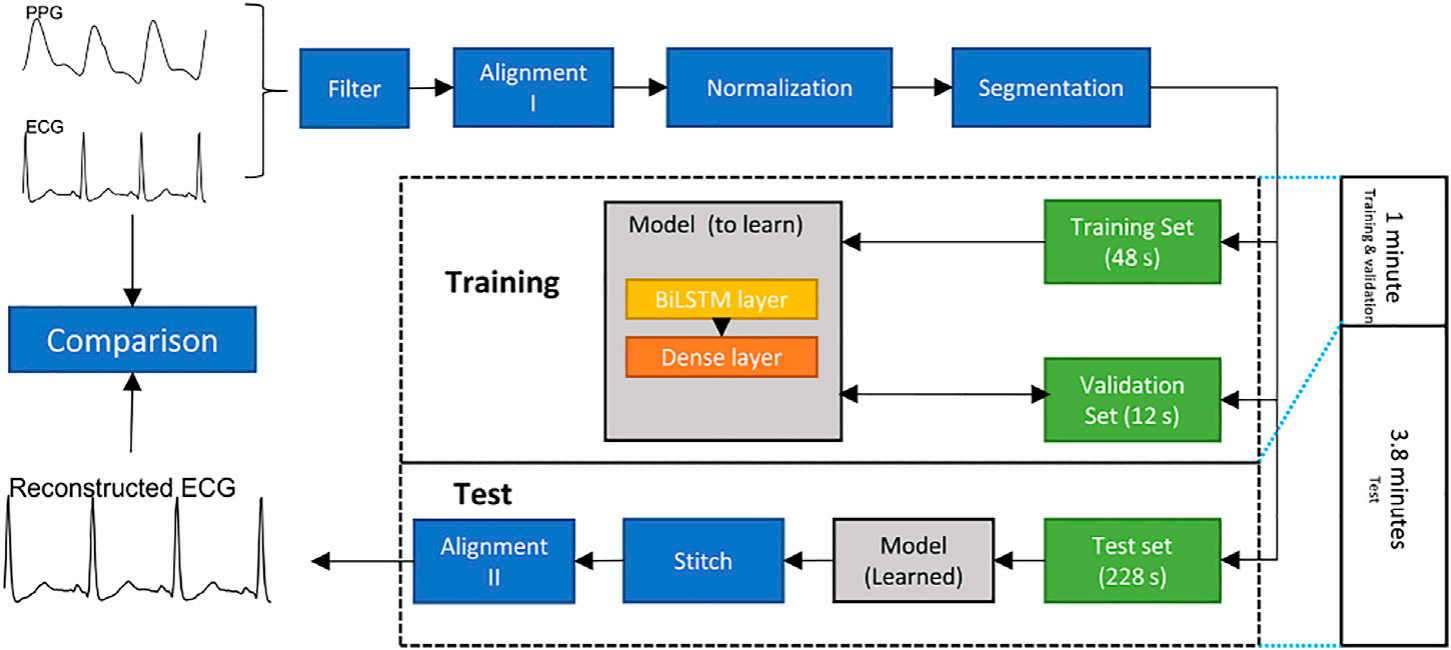
Flowchart of constructing an electrocardiogram (ECG) signal from a photoplethysmogram (PPG) signal. The bidirectional long short-term memory (BiLSTM) model is trained and validated for 1 minute to generate 3.8 min ECG signal.

The overall structure of the proposed method is shown in Figure 2.

## Preprocessing

• **Filter:** Both ECG and PPG signals are filtered by a fourth-order Chebyshev II filter. The frequency range of the filter used in the ECG was 0.5–20 Hz. The frequency range in the PPG was 0.5–10 Hz.
• **Alignment I:** The R peak and systolic peak are the main features in ECGs and PPGs, respectively. For each R peak in an ECG, the corresponding systolic peak in the PPG is the one between the current and next R peaks in the ECG. The Pan-Tompkin method was used to detect the R peaks in ECG, and a block method was used to detect the systolic peaks in the PPG (Pan and Tompkins, 1985; Elgendi et al., 2013). Subsequently, the third systolic peak in the PPG was aligned to the corresponding R peak in the ECG. Note that the third beat was used to ensure that the alignment process was effectively achieved. Sometimes the first PPG beat is not completely clear, and the beat detector cannot detect it, and the second PPG beat could have a correspondence ECG beat that is appearing close to it. This step generated a pair of aligned ECGs and PPGs. Note that the alignment process here means the systolic peak of the PPG beat is exactly aligned with the R peal of the ECG beat.
• **Normalization:** After alignment I, the PPG signals are scaled to [0,1]. Note that the aligned ECG was not normalized so that we can compare the predicted ECG with the reference ECG.
• **Dataset splitting:** To evaluate the long-term performance of the proposed model, the first 60 s of the aligned signal are used to train and validate, while others are used to test. Note that the alignment I step causes the length of the aligned signals to be less than 300 s and the length of each record to be inconsistent. To keep the training data length consistent, we utilized the first 48 s of each record as the training set, the next 12 s as the validation set, and the next 228 s as the test set. Note that there are 12 s ignored to achieve the next step, which discusses the segmentation length, and 12 is found to be the least common that allows the remaining length dividable by 1, 2, 3, and 4.
• **Segmentation:** To make the model faster, a common approach is to segment long signals into segments and process the segments. In this research, the ECG signal was divided according into time segments. The signals in the training, validation, and test sets were divided into segments of the same length, *n* seconds. To find the optimal segment length, *n* = 1, 2, 3, 4 was used to test the model.

### Model Choice

Using the deep neural network is a promising way to achieve our goal, especially recurrent neural networks that can use their internal memory to process a sequential input. We chose bidirectional long short-term memory (BiLSTM) model because it has been successful in solving sequential and time-series problems, such as handwritten recognition (Sakib et al., 2020; Wang et al., 2020). In addition, a study using generative adversarial networks to synthesize ECG showed that the BiLSTM is a robust model for generating ECG signals (Zhu et al., 2019). Note, no other physiological signals were used, only ECG and PPG signals. Between the PPG and ECG, there may be unknown time delays in important events; long short-term memory (LSTM) and BiLSTM are suitable for processing time series in this case. As an extension of the LSTM, the BiLSTM models takes much longer to reach equilibrium than regular LSTM models, but provides better predictions (Siami-Namini et al., 2019).

The model was built based on the TensorFlow package (Version 2.5.0) in Python (version 3.9). The layers of this model included the input layer, BiLSTM layer, fully connected layer. There are further explanations regarding these below.

BiLSTM: BiLSTMs are an extension of traditional LSTMs. BiLSTM connects two LSTM layers of opposite directions to the same output, implying that BiLSTMs train two LSTMs instead of one, on the input sequence, in problems where all time steps are available. With this form of deep generative learning, the output layer can simultaneously get information from past (backward) and future (forward) states (Zhu et al., 2019). In this study, the number of hidden unites in the BiLSTM layer was 25, and the output of the BiLSTM layer is a sequence.

Dense layer: The dense layer is a fully connected layer. In neural networks, a fully connected layer means that all inputs in one layer are connected to each activation unit in the next layer. In machine-learning models, the last layer is usually a fully connected layer to facilitate compiling the data extracted from the previous layer into the final output. The input of the dense layer is the sequence output by the BiLSTM layer, while the output of Dense layer is 1.

Regularization was implemented to solve or prevent the ill-posed problem from overfitting (Bühlmann and van de Geer, 2011). In this research, both Lasso Regression (L1) and Tikhonov regularization (L2) were used in the BiLSTM layer. The kernel regularizer parameter in the BiLSTM layer is L1 = 0.0001, L2 = 0.0001.

The entire model was trained as an option in batch size = 1 and max epochs = 1000. The learning rate is 0.001.

### Stitching the Reconstruction ECG Segments

The output signal length of the model was *n* seconds. To evaluate the long-term performance of the model, we stitched the reconstructed ECG segments together. The reconstructed ECG segments were stitched one by one according to the order of the PPG segments in the test set. Note that the stitching here is carried out by placing segments adjacent to each other.

### Alignment II

The result of stitching is already the reconstructed ECG signal, and this alignment II is mainly to better evaluate the similarity of the reconstructed ECG signal with the reference signal. Note that the alignment II step is based on the cross-correlation.

### Performance Evaluation

Three measures are used to evaluate the performance of the filtered ECG and the reconstructed ECG in the proposed model.

**Pearson**’**s correlation coefficient (** ***r*** **):** This is used to measure the linear correlation between two variables (Liu, 2016). The value of *r* is in the range of [ − 1, 1], where ±1 indicates the strongest possible agreement and 0 indicates the strongest possible disagreement. The formula of *r* is as follows:

$$r=\frac{\sum_{i=1}^{l}\left(ECG_{ref}\left(i\right)-\bar{ECG_{ref}}\right)\sum_{i=1}^{l}\left(ECG_{rec}\left(i\right)-\bar{ECG_{rec}}\right)}{\sqrt{\sum_{i=1}^{l}{\left(ECG_{ref}\left(i\right)-\bar{ECG_{ref}}\right)}^{2}}\sqrt{\sum_{i=1}^{l}{\left(ECG_{rec}\left(i\right)-\bar{ECG_{rec}}\right)}^{2}}}$$ (1)

In this formula, *ECG*_*ref*_(*i*) and *ECG*_*rec*_(*i*) are the individual sample points of the reference ECG and reconstruction ECG indexed with *i*, respectively. The variable *l* is the sample size of the reference ECG. The variables $\bar{ECG_{ref}}$ and $\bar{ECG_{rec}}$ are the mean of sample value of reference ECG and reconstruction ECG, respectively.

**Root mean square error** **(*rmse*)** **:** In machine learning, *rmse* is commonly used to measure the difference between the model’s estimated value and the observed value. The formula of *rmse* is as follows:

$$rmse=\sqrt{\frac{1}{l}\overset{l}{\underset{i=1}{\sum }}{\left(ECG_{ref}\left(i\right)-ECG_{rec}\left(i\right)\right)}^{2}}$$ (2)

**Dynamic time warping (DTW) distance:** DTW can be used to measure the similarity between two time series, which may vary in speed. (Efrat et al., 2007) This method calculates an optimal match between any two given time series. In this paper, the steps to calculate DTW are as follows:

• Suppose the length of the reference ECG is *m*. Create an *m*×*m* matrix. An element *d*_*ij*_ in this matrix is the Euclidean distance between the *i*th sample of reference ECG and the *j*th sample of reconstruction ECG. The formular for calculating *d*_*ij*_ is as follows:

$$d_{ij}=\sqrt{\left({\left(i-j\right)}^{2}+{\left(ECG_{ref}\left(i\right)-ECG_{rec}\left(j\right)\right)}^{2}\right)}$$ (3)

• Looking for the optimal path to minimize the sum of *d*_11_ to *d*_*mm*_ along this path. This path is defined as the warping path, and the sum is the DTW distance.

Similar to *rmse*, the value range of DTW distance is zero to infinity, and the smaller the value, the more similar the two time series are (Efrat et al., 2007).

## Results

**FIGURE 3:**
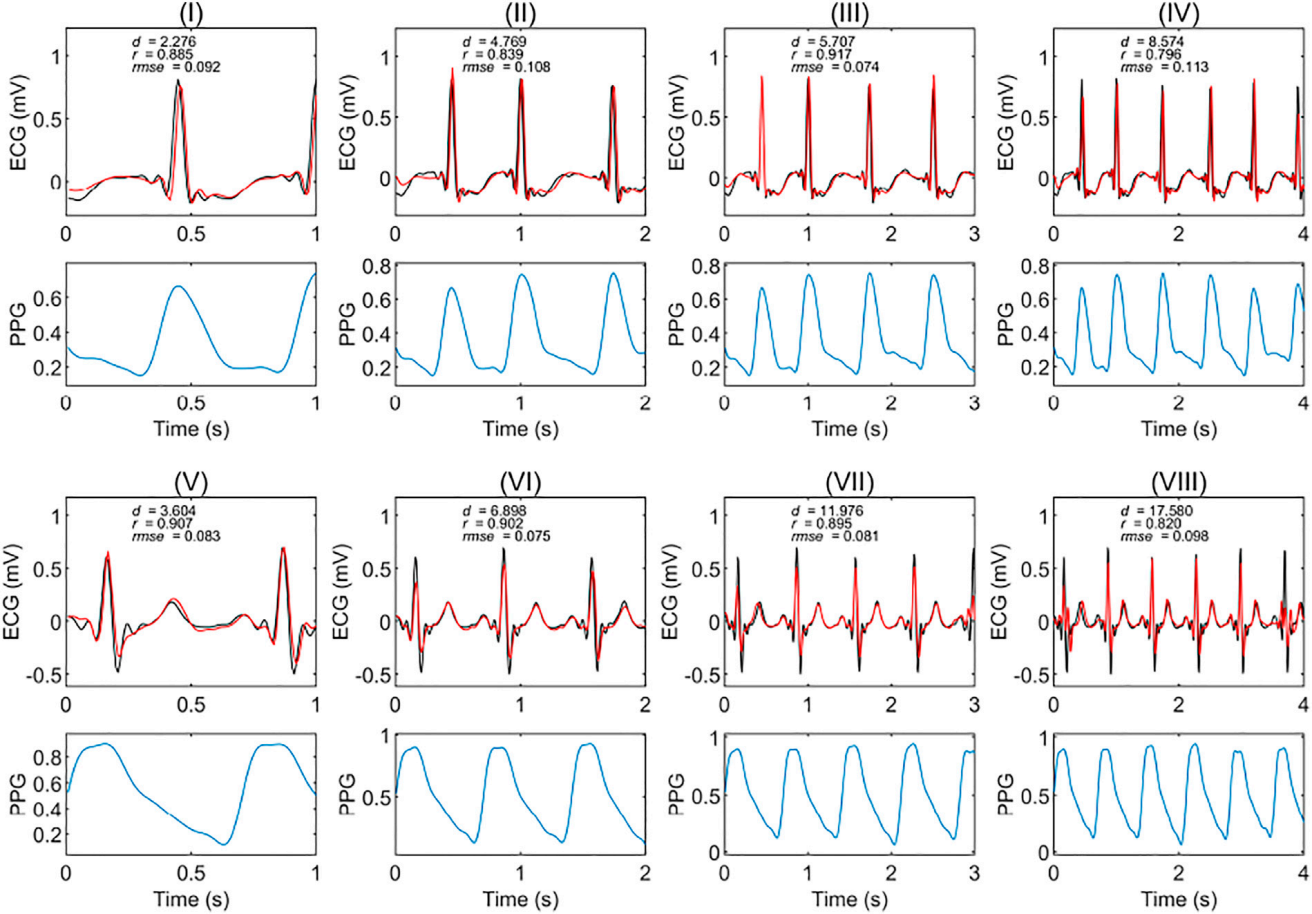
Extracts of experimental results in patients with and without circulatory disease. The red and black curves are the reconstruction ECG and reference ECG, respectively. The abbreviations *r*, *rmse*, and *d* are the Pearson’s correlation coefficient, root mean square error, and dynamic time warping distance, respectively. (I), (II), (III), and (IV) are the results of a subject with a circulatory disease in segments of one, two, three, and 4 seconds, respectively. (V), (VI), (VII), and (VIII) are the results of a subject without any circulatory disease in segments of one, two, three, and 4 seconds, respectively. The PPG is the corresponding segment used to generate the ECG signals.

We tested the model’s accuracy when the data was divided into one, two, three, and four seconds. Figure 3 shows two examples of the experimental results from patients with and without circulatory disease. This figure shows that the ECG reconstructed using different time length models can match the reference ECG. For Figure 3IV, the correlation was only 0.79; however, the morphology of the reconstruction ECG looks highly similar to the reference ECG.

After checking the fitting results of different records, there was a time delay between the reconstruction ECG and the reference ECG for some records. This delay lowered the *r* value; to better evaluate the model’s performance, we used cross-correlation to align the reconstruction ECG with the reference ECG.

**FIGURE 4:**
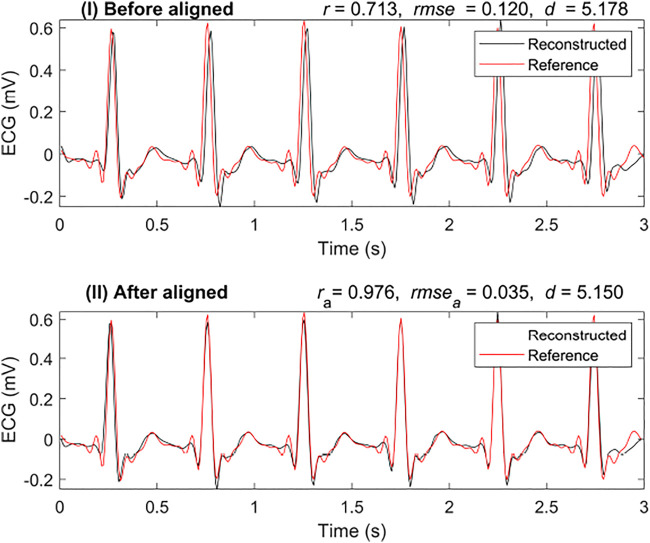
Comparison of reconstruction ECG and reference ECG with and without alignment. The abbreviations *r*_*a*_, *rmse*_*a*_, and *d*_*a*_ are calculated after aligning the reconstruction ECG and reference ECG, respectively.

Cross-correlation can be used to measure the displacement of one time series relative to another of two similar time series. After calculating the cross-correlation between the two time series, the maximum of the cross-correlation function indicated the point at which the signals were best aligned. In this research, we applied cross-correlation with a window of 80 milliseconds. Figure 4 shows a comparison of the results with and without alignment by cross-correlation. It is a segmented result from the 3-s segment-based model. Figure 4I shows the results that are not aligned based on cross-correlation, *r* was 0.713, and *rmse* was 0.120 mV. However, with alignment, as shown in Figure 4II, *r* increased to 0.976 and *rmse* reduced to 0.035 mV. In this case, it was necessary to align the reconstructed ECG with the reference ECG.

To better evaluate the similarity between the two signals, we introduced another similarity measure: DTW distance (*d*). In Figure 4, the DTW distance for the signal with alignment is not very different from that without alignment. The DTW distances of the results were 5.178 mV and 5.150 mV with alignment and without, respectively. This shows that the DTW distance may be a better measure than *r* when testing the similarity in time series with time delay.

**FIGURE 5:**
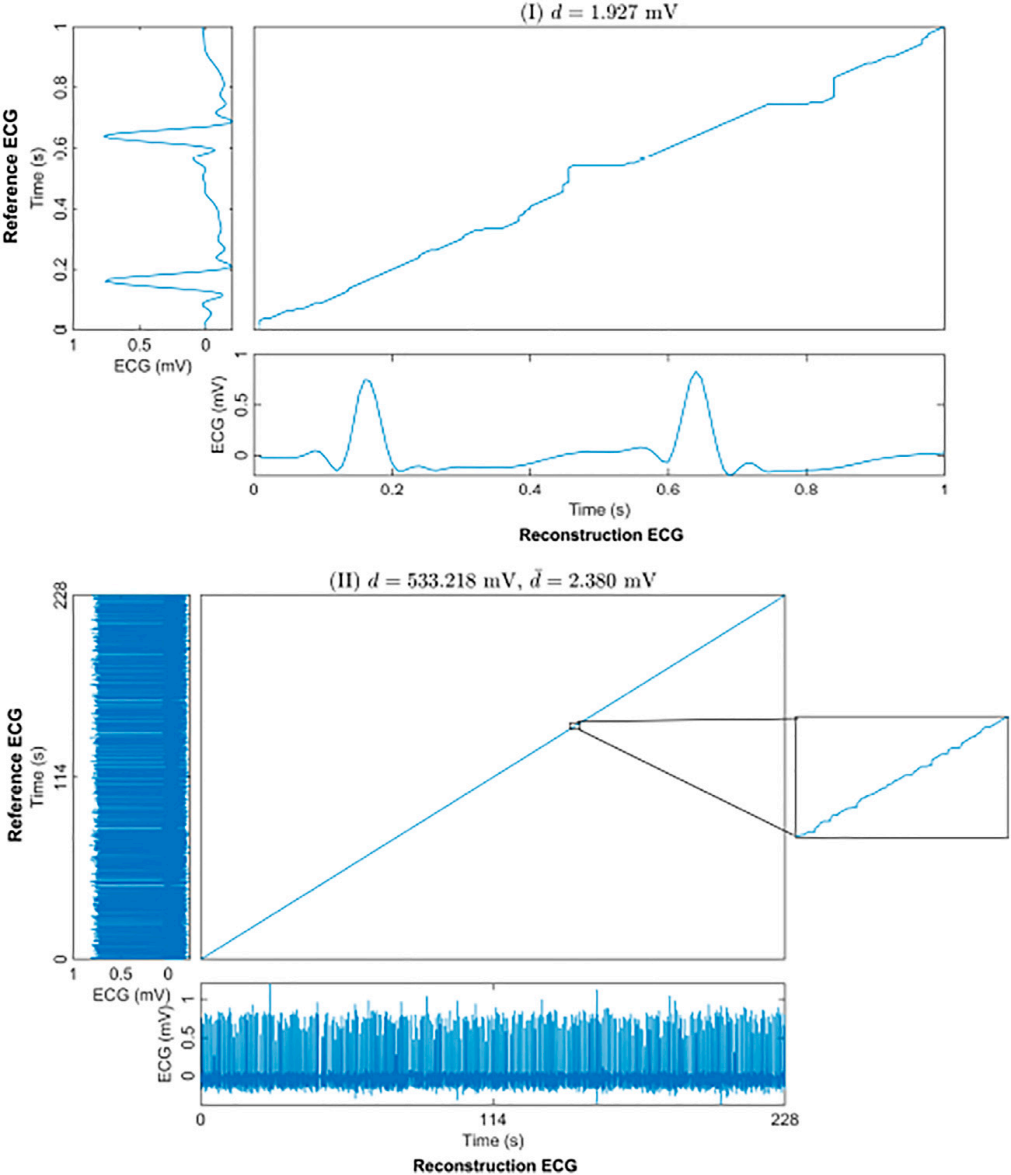
The optimal warping path of the reconstruction and reference ECGs using DTW. (I) shows an example of a 1-s segment. (II) shows the result of the whole 228-s test set for one subject. *d* is the DTW distance of the 228-s reconstruction and reference ECGs. And $\bar{d}$ is *d* divided by the signal time length of 228 s.

The warping path of the DTW can be used as another way to express the similarity of two time series. Figure 5I shows an example of the optimal warping path between the reconstruction ECG and the reference ECG in a 1-s segment-based model. The distance between the reconstruction ECG and the reference ECG was 1.927 mV. The optimal warping path indicated that after a small amount of warping, the reconstructed ECG matched the reference ECG. Figure 5II shows the optimal warping path between the long-term reconstruction ECG and the reference ECG. The long-term reconstruction ECG comes from the result of the 1-s segment-based model after stitching the reconstructed 1-s ECG segments. The distance between the reconstructed and the reference ECG signals is 533.218 mV. Since the DTW distance increases along with the signal length, we divided this distance by the signal time length of 228 s to facilitate a better comparison of the reconstruction results. The average distance was 2.380 mV per second, indicating that, on average, the distance between the two signals per second was minuscule. The warping path of the 228-s reconstruction ECG and the reference ECG appears as a straight line. The magnified portion of the figure shows the warping path of two signals in one second. This means that after a small time shift, the reconstructed ECG can be matched to the reference ECG; that is, there is a high degree of similarity between the two signals.

Table 1 shows the statistical results of the three measures for different signal length models on the 100 records. By comparing the results of the training, validation, and test sets in all the models, the results with alignment decrease more slowly than the results without alignment. In the result of the stitched ECG, the change in DTW distance (*d*_*s*_) was mainly due to the inconsistent time length of each data set. In the average DTW distance $\left(\bar{d_{s}}\right)$ , the downward trend of similarity was consistent with *r*. After comparing the results of the test set of the various time length models, the highest correlation of a single segment was the 1-s segment-based model, and the highest after stitching was the 4-s segment-based model. The stitching step causes the correlation to decrease, which is mainly caused by the inconsistent time delay between the reconstructed ECG and the reference ECG in each segment. By comparing the results of the validation set and test set, we found that the performance of the model degrades on the long training set, but the correlation is still above 0.8.

**TABLE 1:** The results of the test in different lengths. The abbreviations *r*, *rmse*, and *d* refer to the Pearson’s correlation coefficient (*r*), root mean square error *rmse*, and dynamic time warping distance between the reconstruction ECG and reference ECG, respectively. The *r*_*s*_, *rmse*_*s*_ and *d*_*s*_ represent the values between the reconstruction ECG and reference ECG after stitching all the segments together. The abbreviations *r*_*a*_, *rmse*_*a*_, *r*_*sa*_, and *rmse*_*sa*_ is calculated after aligning the reconstruction ECG and reference ECG in the cross-correlation. Finally, $\bar{d}$ and $\bar{d_{s}}$ represent *d* and *d*_*s*_ divided by the signal time length, respectively.

Segment Length (seconds)	Dataset	Number of Segments	***r*(*r***_***a***_ **)**	***Rmse*** (mV) (***rmse***_***a***_ (mV))	***r***_***s***_ **(*r***_***sa***_ **)**	***rmse*_*s*_** (mV) (***rmse*_*sa*_** (mV))	***d*** (mV) **(** $\bar{d}$ (mV))	***d***_***s***_ (mV) **(** $\bar{d_{s}}$ (mV))
*n* = 1	Train	48	0.929 ± 0.095	0.048 ± 0.034	0.926 ± 0.047	0.052 ± 0.027	2.087 ± 1.263	95.569 ± 44.683
			(0.963 ± 0.039)	(0.038 ± 0.024)	(0.928 ± 0.046)	(0.052 ± 0.026)	(2.087 ± 1.263)	(1.991 ± 0.931)
	Validation	12	0.840 ± 0.175	0.071 ± 0.047	0.838 ± 0.108	0.077 ± 0.037	2.319 ± 1.718	26.177 ± 13.084
			(0.945 ± 0.065)	(0.045 ± 0.031)	(0.893 ± 0.066)	(0.063 ± 0.032)	(2.319 ± 1.718)	(2.181 ± 1.090)
	Test	228	0.782 ± 0.222	0.084 ± 0.061	0.774 ± 0.113	0.094 ± 0.046	2.432 ± 1.809	512.876 ± 253.379
			**(0.940 ± 0.074)**	**(0.048 ± 0.042)**	(0.805 ± 0.095)	(0.088 ± 0.045)	(2.432 ± 1.809)	(2.249 ± 1.111)
*n* = 2	Train	24	0.930 ± 0.085	0.048 ± 0.031	0.929 ± 0.054	0.051 ± 0.026	4.109 ± 2.050	95.267 ± 42.718
			(0.956 ± 0.037)	(0.041 ± 0.022)	(0.931 ± 0.052)	(0.050 ± 0.026)	(2.054 ± 1.025)	(1.985 ± 0.890)
	Validation	6	0.809 ± 0.151	0.079 ± 0.046	0.806 ± 0.109	0.083 ± 0.040	4.559 ± 2.313	26.316 ± 11.799
			(0.932 ± 0.064)	(0.050 ± 0.029)	(0.874 ± 0.077)	(0.068 ± 0.033)	(2.280 ± 1.157)	(2.193 ± 0.983)
	Test	114	0.791 ± 0.193	0.083 ± 0.050	0.788 ± 0.096	0.089 ± 0.039	4.643 ± 2.497	502.629 ± 217.656
			(0.924 ± 0.078)	(0.052 ± 0.033)	(0.817 ± 0.071)	(0.084 ± 0.037)	(2.322 ± 1.249)	(2.205 ± 0.955)
*n* = 3	Train	16	0.920 ± 0.101	0.050 ± 0.034	0.919 ± 0.077	0.053 ± 0.030	6.323 ± 3.502	98.667 ± 51.403
			(0.947 ± 0.067)	(0.043 ± 0.026)	(0.920 ± 0.077)	(0.053 ± 0.030)	(2.108 ± 1.167)	(2.056 ± 1.071)
	Validation	4	0.826 ± 0.154	0.079 ± 0.044	0.826 ± 0.122	0.079 ± 0.040	4.559 ± 2.313	26.316 ± 11.799
			(0.917 ± 0.082)	(0.054 ± 0.031)	(0.891 ± 0.091)	(0.063 ± 0.034)	(2.298 ± 1.220)	(2.243 ± 1.154)
	Test	76	0.783 ± 0.199	0.084 ± 0.049	0.788 ± 0.121	0.089 ± 0.038	6.998 ± 3.744	510.932 ± 243.621
			(0.911 ± 0.095)	(0.055 ± 0.034)	(0.813 ± 0.088)	(0.084 ± 0.036)	(2.333 ± 1.248)	(2.241 ± 1.069)
*n* = 4	Train	12	0.926 ± 0.082	0.049 ± 0.030	0.925 ± 0.054	0.052 ± 0.025	7.970 ± 3.926	93.664 ± 42.493
			(0.950 ± 0.043)	(0.043 ± 0.023)	(0.926 ± 0.052)	(0.051 ± 0.025)	(1.993 ± 0.982)	(1.951 ± 0.885)
	Validation	3	0.835 ± 0.140	0.075 ± 0.042	0.834 ± 0.119	0.076 ± 0.039	8.523 ± 4.018	25.015 ± 11.053
			(0.920 ± 0.064)	(0.053 ± 0.030)	(0.904 ± 0.065)	(0.059 ± 0.031)	(2.131 ± 1.005)	(2.085 ± 0.921)
	Test	57	0.792 ± 0.186	0.083 ± 0.048	0.790 ± 0.103	0.088 ± 0.037	8.769 ± 4.330	483.404 ± 207.199
			(0.910 ± 0.086)	(0.055 ± 0.032)	**(0.818 ± 0.072)**	**(0.083 ± 0.035)**	(2.192 ± 1.082)	**(2.120 ± 0.909)**

## Discussion and Limitations

We propose a model based on BiLSTM to reconstruct ECG from PPG. Table 2 shows the comparison between this method and other studies on subject-specific models. The most significant advantage of this model is that it does not rely on period information of the PPG and ECG. There is a difference between the R peak of the ECG and the systolic peak of the PPG, which is called the pulse arrival time, and the existence of the pulse arrival time makes the PPG have a lag relative to the ECG (Liang, 2019). Therefore, the Alignment I step removed pulse arrival times to align the PPG systolic peak with the ECG R peak. After Alignment I, the systolic and diastolic periods are ignored. This Alignment ensures that there is no delay between the peaks. In clinical settings, due to the various factors, such as noise, disease, and other issues, it is difficult and sometimes impossible to extract the periodic information of some PPG and ECG signals. Although some studies have shown that the heart rate variability of photoplethysmography is correlated to that of electrocardiography (Polanía et al., 2015; Weinschenk et al., 2016; Paradkar and Chowdhury, 2017), the RR interval of ECG is not completely consistent with the peak-to-peak interval of PPG, and there is an offset (Luo et al., 2012). The time delay between the reconstruction ECG and the reference ECG may arise from the PPG period information noise and the inconsistency between the ECG and PPG periods. The model proposed herein can solve this problem effectively. As the results show, in the stitched ECG, the performance of the model increases with the segment length. This means that as the segment length increases, the proposed model can work with a small difference between the RR interval and the peak-to-peak interval. Compared to the performance of the model in some of the test sets, we paid greater attention to whether the trained model can be used for long-term construction, i.e., construction of long signals. This is because the long-term performance of the model is important if the technology is to be used in wearable devices. To better evaluate the long-term performance of the model, we did not use the traditional 80*%* training set and 20*%* test set. Instead, 80*%* (48*s*) of the first minute is used for training, and 20*%* (12 s) is used for validation. After the model was trained, we used the next 228 s as the test set. Note that the ratio of the duration of the test set to the training set is 4.75. For short-term performance, the proposed model achieves 0.904 in the 4-s segment-based model (the validation result), which is the same as the beat-based model in the self-collected dataset. The proposed model is only trained on 0.8 min data, while the other two models use more data. Although the performance of the proposed model degrades in the long-term data operation, the correlation still reaches 0.818. These results show that the model we used above can be employed in long-term construction after training.

**TABLE 2:** Comparison of this paper and other papers in the subject-specific model. The ‘NR’ stands for not reported.

	Segmentation Method	Data Used	The Training Segment Length per Subject (minutes)	Performance					
				Test to Training Ratio: 0.25			Test to Training Ratio: 4.75		
				*r*	***rmse*** (mV)	** $\bar{d_{s}}$ ** (mV)	*r*	***rmse*** (mV)	** $\bar{d_{s}}$ ** (mV)
This paper (subject-specific)	Seconds	MIMIC III Johnson, (2016): 100 subjects	0.8	0.904	0.059	2.085	0.818	0.083	2.120
Beat-based model Zhu et al. (2021) (subject-specific)	Beat	TBME-RR Karlen et al. (2013): 42 subjects	6.4	0.984	NR	NR	NR	NR	NR
		MIMIC III Johnson, (2016): 103 subjects	4	0.940	NR	NR	NR	NR	NR
		Self-collected: 2 subjects	24 and 33.6	0.904	NR	NR	NR	NR	NR
XDJDL model Tian et al. (2020) (subject-specific)	Beat	MIMIC III Johnson, (2016): 33 subjects	12	0.88	NR	NR	NR	NR	NR

To evaluate the similarity in the presence of the time delay, we utilized the metric DTW distance. Usually, the warping path of the DTW is used as a measure. The distance is the sum of the cost of DTW along the optimal warping path. However, the DTW distance increases as the signal length increases. Within one second, the average distance for 100 records between the reconstruction ECG of the proposed model and the reference ECG is only 2.120 mV in the 4-s model. The reason behind us using DTW is that the beat intervals of PPG and ECG are not precisely the same. In this paper, Pearson’s correlation coefficient and *rmse* are used to evaluate the linear correlation and the mathematical difference between the reconstructed result and reference ECG, respectively. At the same time, DTW provides another mathematical perspective for evaluating temporal differences, and comparing the difference between the reconstructed result and the reference. By using these three evaluation metrics, we can better evaluate the reconstruction results.

The beat-based model proposed a group model that can work for different subjects. They put those with circulatory system diseases into one group and those without circulatory system diseases into another group. In this study, we did not train based on subject grouping. Electrocardiography and photoplethysmography reflect the electrophysiological and mechanical activities of the heart, respectively. The excitation-contraction coupling suggests that the electrical signals of the heart trigger the heart to have periodic contractions. If, from a signal-processing perspective, the heart and blood transmission system are like a black box, the ECG signal is the input of the black box, and the PPG signal is the output. For each individual, there may be differences in their transfer functions due to various factors, especially in the presence of circulatory diseases. Therefore, in this study, we only considered subject-specific models.

The limitation of this paper is that the frequency range of the reconstructed ECG is below 20 Hz. However, the frequency range of abnormal ventricular conduction is above 70 Hz (Tereshchenko and Josephson, 2015). This means that the model cannot reconstruct the abnormal ventricular conduction in an ECG, a primary reason being that the commonly used frequency range in PPG is 0.5–20 Hz. This paper focuses on the commonly used frequency range in PPG. In future work, we will test the performance of this model in the different frequency ranges. It is worth noting that the purpose of this study is to develop a subject-specific model. The next step would be to examine its performance for the intra-subject case against the inter-subject performance case.

For subjects with different circulatory system diseases, the shape of their ECGs may deviate from that of the normal ECG in various ways. Therefore, using the same model to reconstruct ECGs from PPGs for multiple subjects is a challenge. Thus, to use this model effectively, abnormalities in an ECG need to be extracted from the PPG. At present, studies have shown that some arrhythmia can be detected by a PPG (Polanía et al., 2015; Paradkar and Chowdhury, 2017), but these still depend only on the correlation between the ECG and the PPG on the beat. Other deep learning algorithms with different parameter choices need to be investigated in future work. It is also desirable to carry further evaluations of the differences between the reconstructed and reference ECG features, such as QRS complex and ST segment, in future studies.

## Conclusion

This study proposed a subject-based model to reconstruct an ECG from photoplethysmography. The model was trained on short ECG and PPG signals (0.8 min), but it successfully reconstructed long (3.8 min) ECG signals. For each segment of the constructed ECG, the average correlation between the constructed and the reference ECG was 0.94 when the segments were 1-s long. For the entire test ECG, the average correlation of the 4-s segment-based model was 0.91. Compared to other models, this model divides the PPG into segments of seconds (instead of beats) to generate ECG segments and solve the inconsistency between ECG and PPG beats in some signals. The long-term reconstructed ECG is highly similar to the waveform of the reference ECG. This model is expected to be used in wearable devices as an effective alternative for a low-cost, long-term health or fitness monitoring application.

## Data Availability

Data and code used in this article can be downloaded from this link https://github.com/Elgendi/PPG2ECG.
